# Ramadanov–Zabler Safe Zone for Sacroiliac Screw Placement: A CT-Based Computational Pilot Study

**DOI:** 10.3390/jcm14103567

**Published:** 2025-05-20

**Authors:** Nikolai Ramadanov, Simon Zabler

**Affiliations:** 1 Center of Orthopaedics and Traumatology, Brandenburg Medical School, University Hospital Brandenburg an der Havel, Brandenburg an der Havel, 14770 Brandenburg, Germany; 2 Faculty of Health Science Brandenburg, Brandenburg Medical School Theodor Fontane, Brandenburg an der Havel, 14770 Brandenburg, Germany; 3 Faculty of Applied Computer Science, Deggendorf Institute of Technology, 94469 Deggendorf, Germany

**Keywords:** pelvic fractures, sacroiliac joint/surgery, bone screws, fracture fixation, internal, tomography, X-ray computed, three-dimensional imaging

## Abstract

**Background/Objectives**: Posterior pelvic ring fractures are severe injuries requiring surgical stabilization, often through sacroiliac (SI) screw fixation. However, improper screw placement poses risks of neurovascular injury and implant failure. Defining a precise safe zone for screw placement is crucial to improving surgical accuracy and reducing complications. **Methods**: A computational study was conducted using a CT scan of a 75-year-old male patient to establish a safe zone for SI screw placement. Manual segmentation and 3D modeling techniques were used to analyze bone density distribution. A 2D lateral projection of the sacrum was generated to identify high-density regions optimal for screw placement. While the general principle of targeting areas of higher bone density for screw insertion is well established, this study introduces a novel computational method to define and visualize such a safe zone. The resulting region, termed the Ramadanov–Zabler Safe Zone, was delineated based on this analysis to ensure maximal intraosseous fixation with minimal risk of cortical breaches. **Results**: A high-resolution 3D model of the sacral region was successfully generated. Standard thresholding methods for segmentation proved ineffective due to low bone density, necessitating a freehand approach. The derived 2D projection revealed regions of higher bone density, which were defined as the Ramadanov-Zabler Safe Zone for screw insertion. This zone correlates with areas providing the best structural integrity, thereby reducing risks associated with screw misplacement. Additionally, intraoperative and postoperative imaging from a representative case is included to illustrate the translational feasibility of the proposed technique. **Conclusions**: The Ramadanov–Zabler Safe Zone offers a reproducible, CT-based computational approach to guide for SI screw placement, enhancing surgical precision and patient safety. This CT-based computational approach provides a standardized reference for preoperative planning, minimizing neurovascular complications and improving surgical outcomes. This pilot technique is supported by preliminary clinical imaging that demonstrates feasibility for intraoperative application. Further validation across diverse patient populations is recommended to confirm its clinical applicability.

## 1. Introduction

Posterior pelvic ring fractures are severe injuries that often result from high-energy trauma [1]. These fractures involve the sacrum, sacroiliac (SI) joint, or iliac wing and can lead to significant instability, pain, and impaired load-bearing capacity. Given their biomechanical importance, surgical stabilization is frequently required to restore pelvic integrity and allow early mobilization. One of the most common techniques for stabilizing posterior pelvic ring fractures is SI screw fixation [2,3]. This method provides minimally invasive stabilization by placing screws across the SI joint or into the sacral body. When properly positioned, SI screws offer strong fixation with minimal soft tissue disruption. However, SI screw placement carries the risk of injuring critical anatomical structures [3,4,5]. The sacral nerve roots, iliac vessels, and surrounding soft tissues are in close proximity to the screw trajectory. Misplacement can lead to neurological deficits, vascular injury, and implant failure, making precise positioning essential. To minimize complications, it is crucial to define a safe zone for SI screw placement. A well-defined safe zone allows surgeons to maximize fixation strength while reducing the risk of iatrogenic injury.

The literature on percutaneous SI screw fixation has evolved significantly over the years, with research focusing on aspects such as postoperative evaluation, screw placement accuracy, and the implementation of advanced imaging and navigation technologies. A 2022 study by Chen et al. [6] compared free-hand versus targeting device placement of posterior SI screws without fluoroscopy. The device showed superior accuracy, with free-hand screws risking neurovascular injury. Key risk areas were identified, but limitations include a small cadaveric sample, no fluoroscopy, and limited clinical validation. A 2005 study by Hilgert et al. [7] evaluated SI screw fixation using fluoroscopy. Proper screw entry is crucial to avoid malpositioning. Postoperative CT of 24 screws showed no errors. Limitations include a small sample size, no control group, and reliance on surgeon experience, affecting reproducibility and comparison with advanced imaging techniques. A 2007 study by Tosounidis et al. [8] analyzed 73 SI screws placed in 41 pelvic ring injuries by seven surgeons using a standardized technique. While no infections or major complications occurred, two malpositioned screws required revision. Screw loosening and bending occurred in three cases. Success depended on surgeon experience, reduction quality, and radiologic visualization. A 2014 study by Tejwani et al. [9] evaluated CT accuracy in assessing reduction, neurologic risk, and revision need after percutaneous SI screw fixation in 46 patients. Foramen penetration occurred in 23 of 51 screws but correlated with neurologic deficit only if >2.7 mm. Limitations include retrospective design and variable injury patterns. CT is recommended selectively. A 2015 study by Shrestha et al. [10] evaluated percutaneous SI screw fixation under single fluoroscopy in 21 patients with sacral fractures or SI joint disruptions. While outcomes were mostly good, complications included DVT, hematoma, and two juxta-foraminal screws. Limitations include a small sample size and retrospective design. Accurate imaging is crucial for safety. A 2017 study by Herman et al. [11] assessed fluoroscopic accuracy in sacral screw placement using postoperative CT and a mathematical safe zone calculation. Among 156 screws in 94 patients, 32% had cortical breaches. The mathematical method improved accuracy (92.9%) over lateral views (70%). Limitations include retrospective design and reliance on imaging rather than clinical outcomes. A 2021 study by McLaren et al. [12] analyzed angular tolerance for SI screw placement using 433 pelvic CTs. S1 and S2 corridors had narrow safe zones (1.53° and 1.02° tolerance). Around 31.1% of patients lacked a viable corridor. Limitations include reliance on CT mapping without clinical validation. A 2022 study by Yang et al. [13] evaluated TiRobot-assisted percutaneous SI screw fixation in seven patients with sacral variations. All fractures healed, and screw placement was highly accurate. However, limitations include the small sample size, short follow-up, and retrospective design. While results were promising, broader studies are needed to confirm safety and efficacy. A 2024 cadaveric study by Aregger et al. [14] assessed fluoroscopic landmarks for SI screw placement. Of 14 pelves, 10 had a viable screw corridor, while 4 posed high risk. A safe zone was defined using the S1 body diagonal. Limitations include a small sample size and lack of CT validation.

CT-based 3D reconstruction relies on segmentation techniques that distinguish bone from surrounding tissue based on Hounsfield Unit (HU) values. Bone structures are typically segmented using threshold ranges that isolate high-density voxels, with cortical bone commonly above +300 HU and trabecular bone in lower ranges. While this approach is widely used, its accuracy can be limited in patients with low bone density or indistinct cortical boundaries, requiring manual correction or refinement. As described by Moldovan et al. [15], segmentation plays a critical role in building patient-specific 3D models and aligning anatomical structures for surgical planning. In this study, segmentation was initially performed using HU thresholding but required manual adjustments to ensure accurate delineation of sacral boundaries due to low bone density in the selected patient.

The need for a well-defined safe zone for SI screw placement arises from the complex anatomy of the pelvic region and the proximity of critical neurovascular structures. Without precise guidance, the risk of intraoperative complications such as nerve injury, vascular damage, or implant failure increases significantly. Defining a safe zone using a 3D model derived from pelvic CT scans offers surgeons a reliable reference for screw placement, reducing variability and improving accuracy. This approach allows for safer and more effective fixation, ensuring optimal outcomes and minimizing the potential for iatrogenic harm during surgery.

The aim of this pilot study was to explore a technique for defining a patient-specific safe zone for SI screw placement using a 3D model derived from a pelvic CT scan. Based on data from a single 75-year-old male patient, this proof-of-concept study seeks to provide surgeons with a reliable reference during percutaneous SI screw fixation and includes a preliminary clinical example to support its practical relevance. By utilizing this model, the study aims to minimize the risk of neurovascular injury and other complications, ultimately improving surgical outcomes and enhancing the safety of the procedure.

## 2. Materials and Methods

A patient who received a pelvic CT scan during an emergency department visit for an unrelated clinical indication provided written informed consent for participation in this study. Approval from the Data Protection Officer of the University Hospital Brandenburg (No. 20250327) was granted and is provided in the Supplementary Materials, including a redacted copy of the original German consent form with all patient-identifying data removed for privacy reasons, as well as an English translation (Supplementary Documents S1–S4). A native CT scan (DICOM format) of a 75-year-old male patient, dated 2 February 2025, was acquired for the purpose of this study. The CT scan revealed no relevant bony anomalies, pathologies, or other abnormalities in the pelvic region. The dataset was subsequently processed and analyzed through a series of computational steps:

1. CT Data Acquisition: The original CT scan dataset was provided for processing and analysis. The scan quality was sufficient, showing no relevant bony deformities or pathological changes in the sacrum and SI region, allowing for accurate 3D modeling.
2. Manual Segmentation: Initial segmentation of the SI region was performed manually using Avizo software (version 2020.3, Thermo Fisher Scientific, Waltham, MA, USA). Standard thresholding techniques were attempted; however, due to the relatively low bone density in the SI region, these methods were ineffective in accurately delineating the joint.
3. Freehand Segmentation: To overcome the limitations posed by the low-density bone structure, a freehand segmentation approach was employed. This method, which involved manually tracing the boundaries of the SI region, took approximately one hour to complete. The result was a high-precision binary mask of the SI region, which was then used to create an accurate 3D model of the joint.
4. 2D Projection Creation: From the 3D model, a 2D lateral view of the sacrum was generated by summing the *Y*-axis slices of the dataset. This 2D projection effectively reflected the bone density distribution within the sacrum and was crucial for visualizing regions of higher bone density. These areas were considered optimal for screw placement, as higher density regions are more likely to provide stable, intraosseous fixation.
5. Generation of Screw Trajectory Target Zone: Using the 2D projection, a patient-specific safe zone for screw placement was identified through a reproducible computational technique developed in this study. The resulting zone is referred to as the Ramadanov–Zabler Safe Zone for reference purposes. Regions with higher bone density were targeted as they suggested a greater likelihood that the screw would remain strictly intraosseous, thereby reducing the risk of cortical breaches or screw misplacement. The target zone served as a guide for the ideal screw trajectory during percutaneous SI screw fixation.
6. To support the translational relevance of the proposed technique in a clinical illustration case, we included intraoperative and postoperative imaging from a representative patient with a B-type pelvic injury. The patient underwent standard percutaneous sacroiliac screw fixation. A lateral fluoroscopic image was obtained intraoperatively after guidewire placement, and postoperative CT scans (sagittal and axial) were acquired routinely.

This entire process aimed to define a precise, image-based safe zone for SI screw placement using a novel CT-based segmentation and projection technique, resulting in what we term the Ramadanov–Zabler Safe Zone.

## 3. Results

### 3.1. Three-Dimensional Model Creation and Segmentation

**Figure 1:**
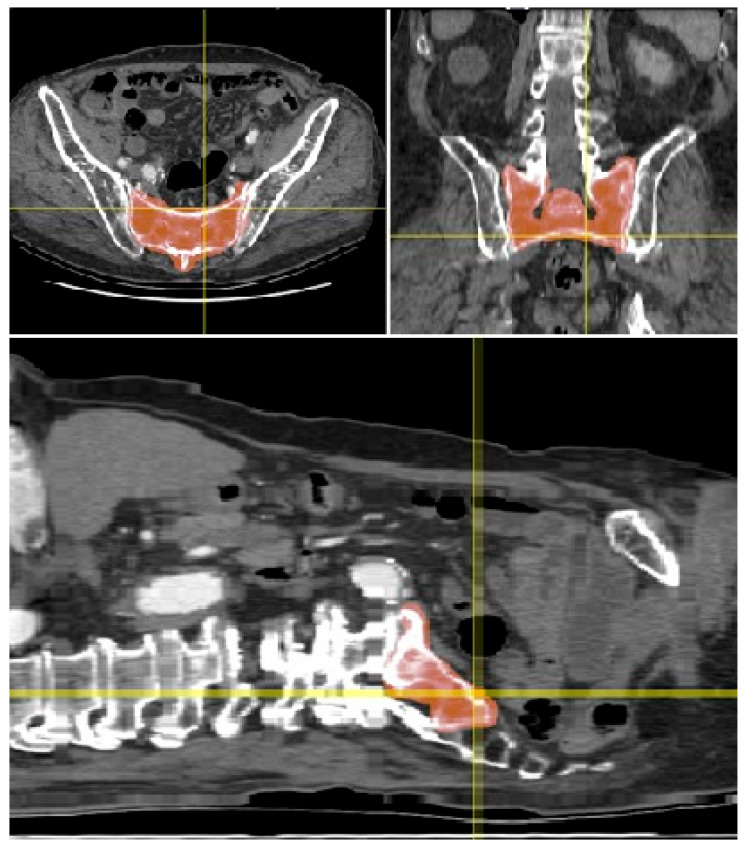
Three orthogonal section views of the patient abdominal multislice (MS) CT scan showing the sacrum (highlighted in red; plane coordinates are marked by the crosshairs). The transverse pixel sampling was 0.672 mm and the longitudinal z-spacing was 2.976 mm.

**Figure 2:**
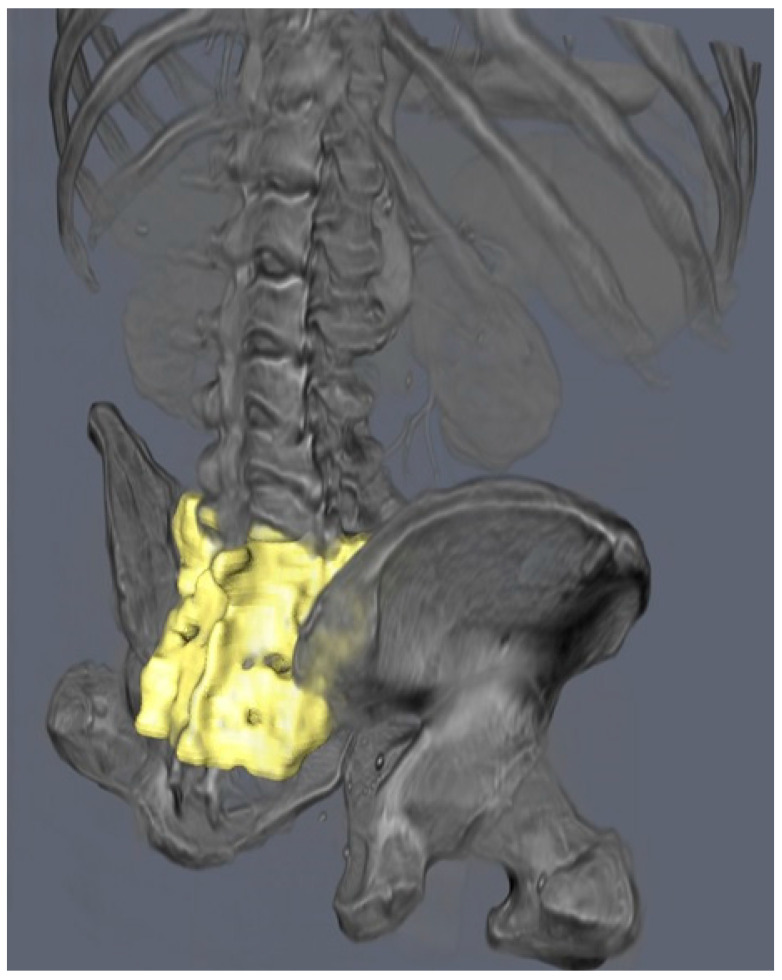
Three-dimensional semi-transparent rendering of the abdominal MSCT scan showing the skeleton’s radio-optical density in grey. The sacrum is converted to an opaque surface mesh after freehand segmentation: the rendering is showing it in bright yellow color.

A high-resolution 3D model of the SI region was successfully generated using the CT scan of a 75-year-old male patient. The original CT dataset, free of any relevant bony anomalies or pathological changes, allowed for precise segmentation of the SI region (Figure 1). Due to the relatively low bone density in the SI region, the initial segmentation methods using standard thresholding were not effective. However, the freehand segmentation approach, which involved manually tracing the boundaries of the SI region, resulted in a high-precision binary mask of the sacrum (Figure 2). This mask was subsequently used to generate an accurate 3D model, which was then subjected to further analysis.

### 3.2. Two-Dimensional Projection Analysis

**Figure 3:**
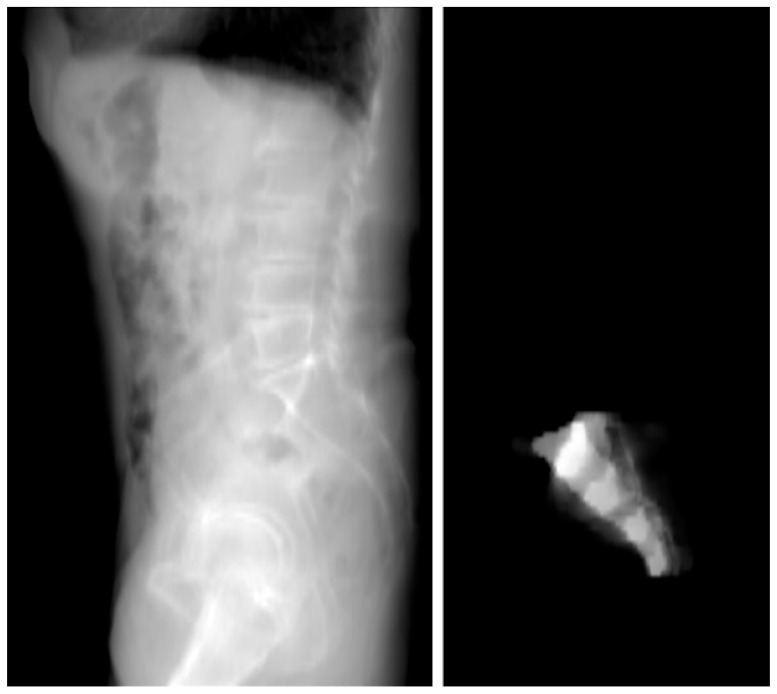
(**Left**): Virtual mediolateral projection of the patient’s radio-optical density (MSCT). (**Right**): Virtual mediolateral projection of the patient’s radio-optical density after masking the sacrum, i.e., eclipsing everything outside the mask. Note, the image contrast and brightness are raised for showing the density differences between the sacral joints.

**Figure 4:**
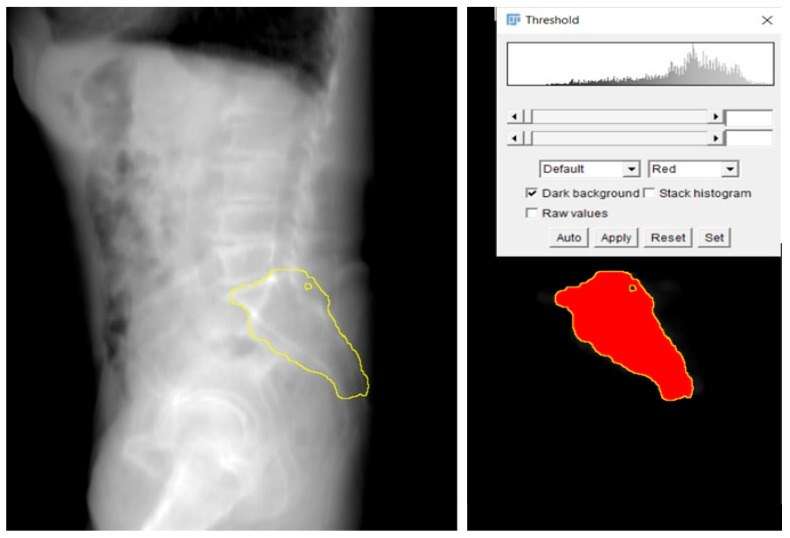
Designing the sacrum contour line by applying threshold segmentation to the masked projection (**right**) and transferring the outline onto the complete scan’s projection (**left**).

From the 3D model, a 2D lateral view of the sacrum was created. Figure 3 shows a 2D view from the sum of the CT slices (Figure 3). This projection reflected the bone density distribution within the sacrum, which helped identify regions with higher bone density, considered optimal for screw placement. The 2D projection allowed for a clearer visualization of these areas and highlighted potential zones where screw placement could offer stable fixation. The threshold is applied to the masked summation image, resulting in a contour line (Figure 4).

### 3.3. Safe Zone Identification

**Figure 5:**
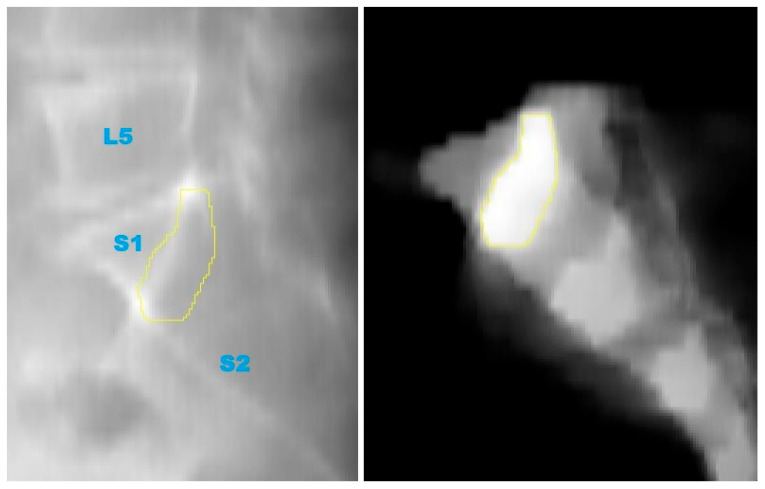
Definition of the safe zone by choosing a threshold high enough to only outline the high density of S1 in the masked projection (**right**) and transferring the outline to the (**left**). L5: fifth lumbar vertebra; S1: first sacral vertebra; S2: second sacral vertebra.

The key step in this study was the identification of the Ramadanov–Zabler Safe Zone for SI screw placement. Based on the 2D projections, the areas with higher bone density were delineated as the target zones for screw insertion (Figure 5). These regions were selected because higher bone density correlates with a greater likelihood that the screw will remain within the sacral vertebra body, thus reducing the risk of cortical breaches, screw misplacement, or neurovascular injury. It is important to note that the body of sacral vertebra 1 is not perfectly shaped, and the lateral X-ray projection represents a summation view of the sacral region. This means that areas with higher bone density in the lateral projection are more likely to correspond to regions with greater structural integrity, offering a more secure pathway for screw insertion. Consequently, these denser areas are considered optimal for screw placement, as they minimize the likelihood of the screw exiting the sacral body and causing complications.

### 3.4. Preliminary Clinical Illustration

**Figure 6:**
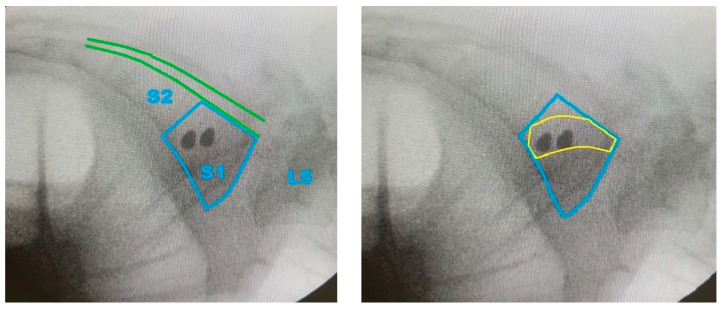
Intraoperative lateral fluoroscopy confirms guidewire placement within the proposed Ramadanov–Zabler Safe Zone (highlighted in yellow). The two guidewires are so well aligned that they appear almost point-like in the lateral view. The blue contour outlines the body of S1, while the green lines indicate the margins of the spinal canal. L5: fifth lumbar vertebra; S1: first sacral vertebra; S2: second sacral vertebra.

**Figure 7:**
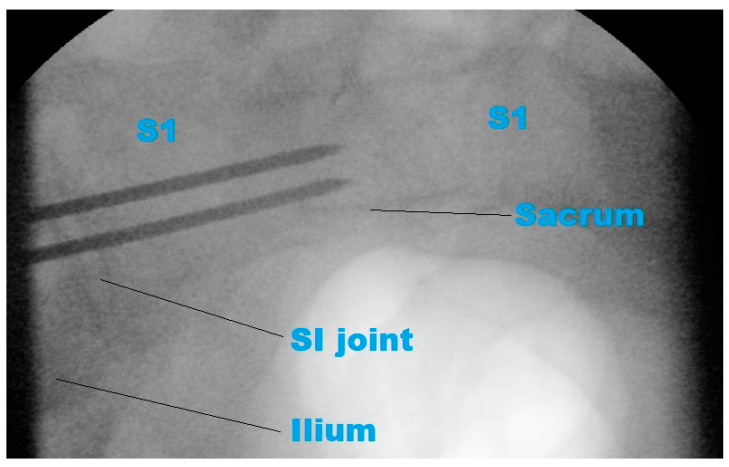
Fluoroscopic inlet view confirms correct guidewire placement in the second plane. S1: first sacral vertebra; SI: sacroiliac.

In the representative case, intraoperative fluoroscopy confirmed guidewire placement aligned with the proposed Ramadanov–Zabler Safe Zone (Figure 6). The fluoroscopic inlet view confirmed that the two guidewires were correctly placed in the second plane (Figure 7). Postoperative axial and sagittal CT images showed that the final screw trajectory remained intraosseous and consistent with the CT-defined safe zone (Supplementary Videos S1 and S2).

## 4. Discussion

The creation of the Ramadanov–Zabler Safe Zone for SI screw placement marks a significant contribution to orthopedic surgery, offering a more precise approach to posterior pelvic ring fracture stabilization. These fractures, often caused by high-energy trauma, present serious challenges due to the proximity of critical neurovascular structures and the complexity of pelvic anatomy. Therefore, precise screw placement is essential to avoid complications such as neurovascular injury, vascular damage, and screw misplacement, which can result in significant morbidity.

Although various methods have been developed to improve the safety of SI screw placement, such as fluoroscopy and freehand techniques, these approaches often have limitations in terms of accuracy and surgeon experience. The Ramadanov–Zabler Safe Zone, derived from a detailed 3D model based on CT scans, aims to overcome these limitations by providing a more accurate reference for screw placement. This zone highlights regions of higher bone density in the sacrum, ensuring that screws are more likely to remain strictly within the sacral body, reducing the risk of cortical breaches or screw misplacement. The concept of defining a safe zone based on bone density distribution in 2D projections is a key advancement that enhances the precision of SI screw placement. The designation “Ramadanov–Zabler Safe Zone” is used here to refer specifically to the zone as defined by the computational technique developed in this study. It serves as a reference term for future validation and discussion of the method.

While the biomechanical rationale for targeting regions of higher bone density during SI screw placement is well established in the literature, this study introduces a specific technique for visualizing and delineating those areas using CT-based 3D modeling and 2D projection. The novelty lies not in the underlying principle, but in the reproducible method of identifying a patient-specific safe zone through computational segmentation. The term “Ramadanov–Zabler Safe Zone” refers to this technique-derived zone, which can serve as a reference in surgical planning.

When compared to relevant literature, the Ramadanov–Zabler Safe Zone presents several key differences. Earlier research, such as that by Hilgert et al. (2005) [7], and Aregger et al. (2024) [14], demonstrated the utility of targeting devices and fluoroscopy for screw placement. However, these methods often relied heavily on surgeon experience and had limitations related to imaging resolution and intraoperative reproducibility [7,14]. The Ramadanov–Zabler Safe Zone proposes a CT-derived approach that defines patient-specific safe zones in a standardized, data-driven manner. Prior CT-based studies, such as those by Tejwani et al. (2014) and Shrestha et al. (2015), assessed screw placement accuracy but were constrained by small sample sizes, inconsistent methodology, and the absence of a clearly delineated safe zone [9,10]. In contrast, the present study introduces computational modeling to address these gaps, offering a more consistent and reproducible framework for accurate screw placement planning.

Additionally, recent studies have introduced geometry- and population-based methods to enhance screw safety. Herman et al. [11] proposed a mathematically derived safe zone based on pelvic inlet and outlet fluoroscopic views, designed to simplify intraoperative decision-making through screw position estimations relative to visible bony landmarks. While effective for real-time use, their approach does not account for individual anatomical variation or bone quality. McLaren et al. [12], in contrast, analyzed CT scans from 433 patients to quantify angular tolerances and osseous corridor diameters for SI screw placement. Their findings—such as angular tolerances of 1.53° for S1 and 1.02° for S2, and the absence of a viable corridor in approximately 31% of patients—offered important anatomical insights but remain rooted in population averages.

The Ramadanov–Zabler Safe Zone builds upon these foundational efforts by introducing a computationally defined, density-based model derived from individual CT data. Rather than relying on geometric boundaries or anatomical corridor statistics alone, this approach visualizes regions of high intraosseous bone density to define an optimal screw trajectory. As such, it offers a personalized, biomechanically informed, and reproducible planning tool that complements existing geometric and anatomical models. This density-driven methodology enhances preoperative planning precision and holds potential for integration with intraoperative navigation or robotic systems.

The Ramadanov–Zabler Safe Zone directly addresses several of the key risks associated with sacroiliac screw placement, including cortical breach, nerve root injury, and vascular damage. By defining a trajectory through high-density intraosseous regions using patient-specific CT data, the zone reduces the likelihood of the screw penetrating the sacral foramen or exiting the cortical bone. This approach minimizes the chance of neurological complications and enhances mechanical anchorage by ensuring better bone purchase. The safe zone thus improves both procedural safety and implant stability compared to techniques that rely solely on anatomic averages or 2D fluoroscopic views.

**Figure 8:**
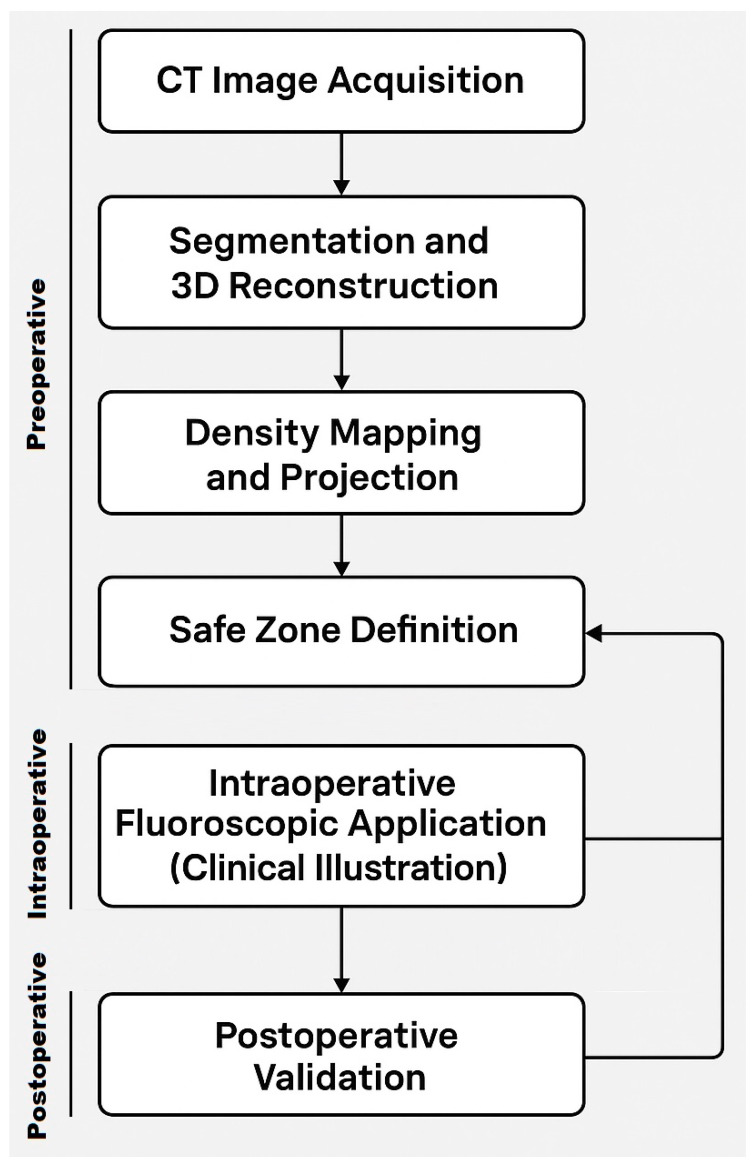
Workflow for defining and applying the Ramadanov–Zabler Safe Zone. The process includes CT acquisition, 3D reconstruction, density mapping, and intraoperative application in a representative clinical case.

To improve transparency and reproducibility, we have outlined a structured workflow (Figure 8) summarizing the steps followed in this study—from CT acquisition and segmentation to intraoperative alignment with the proposed safe zone. While this workflow is modest and specific to the computational scope of this pilot project, it may serve as a practical reference in selected clinical scenarios. Unlike the comprehensive surgical workflow presented by Solyom et al. [16], our model focuses on imaging-based planning and safe zone identification, which could be integrated into preoperative assessment protocols in the future.

### 4.1. Limitations and Strengths

This study has several limitations that should be considered: (i) This study is based on a single CT scan of a 75-year-old male patient. Significant anatomical and biological differences—including sex-based variation, age-related bone density changes, and pathological deformities—could influence the geometry and density of the sacrum, limiting the generalizability of the findings to a broader patient population. (ii) The manual segmentation method introduces potential subjective errors and might be less precise than automated techniques, which could affect the reproducibility of the results. (iii) The results are based on a single data source, and further validation is required to confirm the accuracy and applicability of the model. (iv) Although the segmentation process in this study was performed manually, preliminary evidence suggests it is reproducible. To assess consistency, the segmentation and safe zone identification were perfomed by the senior author (S.Z.) and repeated by the primary author (N.R.) with a two-month interval between sessions. Despite this temporal and observer separation, the resulting safe zones were consistent in shape, location, and extent. While this informal test supports low inter- and intra-observer variability, we acknowledge that a formal quantitative analysis using similarity metrics (e.g., Dice coefficient or Jaccard index) would strengthen the methodological validation and is planned for future work.

Despite these limitations, this study also has several strengths: (i) This study utilizes a CT-derived 3D model, allowing for a precise visualization of the anatomy and better planning of screw placement. (ii) This study introduces a novel approach by developing a safe zone for SI screw placement based on CT data, advancing surgical planning in this domain. (iii) By utilizing the Ramadanov–Zabler Safe Zone, the risk of neurovascular complications is reduced, enhancing patient safety.

### 4.2. Suggestions for Future Research

Future studies should investigate how well the identified Ramadanov–Zabler Safe Zone works in broader clinical and surgical settings, including evaluation across different patient groups. Future research should examine a variety of pelvic anatomies across sexes, age groups, and patients with anatomical or pathological variations (e.g., osteoporosis, sacral dysplasia, trauma-related deformities) to validate the results for a wider patient population. A formula or algorithm that allows for the application of the Ramadanov–Zabler Safe Zone to current intraoperative lateral X-ray projections could significantly enhance clinical applicability and improve precision during surgery. Further research should explore how robotic systems can assist in the precise placement of screws within the Ramadanov–Zabler Safe Zone, improving surgical outcomes. To confirm the actual safety and effectiveness of the Ramadanov–Zabler Safe Zone, long-term studies that assess postoperative complications and surgical outcomes should be conducted. Multicenter clinical studies using diverse patient datasets may further enhance the reproducibility and clinical relevance of this approach. Additionally, future studies should incorporate finite element analysis (FEA) to simulate biomechanical loading of sacroiliac screws placed within the Ramadanov–Zabler Safe Zone. This would help evaluate the structural integrity and mechanical safety of the proposed trajectory under physiological conditions, further validating the technique for clinical use.

### 4.3. Pilot Application in Surgery

To demonstrate the practical feasibility of the proposed technique, intraoperative and postoperative imaging from a representative patient with a B-type pelvic ring injury was included. During standard percutaneous sacroiliac screw fixation, the guidewire was placed under fluoroscopic control and intentionally aligned with the CT-defined Ramadanov–Zabler Safe Zone. Fluoroscopic images captured intraoperatively show the guidewire trajectory matching the projected safe zone.

Postoperative CT scans (sagittal and axial views) confirmed that the final screw trajectory remained intraosseous and corresponded closely with the delineated high-density region. Although this single case does not constitute formal clinical validation, it provides an initial illustration of how the computational technique can be translated into surgical planning and execution. This example supports the translational potential of the Ramadanov–Zabler Safe Zone and offers a foundation for future clinical application and evaluation.

## 5. Conclusions

The Ramadanov–Zabler Safe Zone represents a pilot-stage technique based on CT-derived 3D modeling and computational segmentation to guide precise SI screw placement. While the underlying principle of targeting high bone density is well established, this study introduces a reproducible method for visualizing and delineating a patient-specific safe zone. Tested on a single case, this technique may help minimize complications such as neurovascular injury and implant failure, ultimately contributing to improved surgical accuracy and patient safety. Further research is necessary to validate the technique across broader patient populations and to assess its clinical applicability. Nonetheless, the Ramadanov–Zabler Safe Zone offers a promising tool for preoperative planning in sacroiliac screw fixation, with preliminary imaging support demonstrating clinical feasibility.

## Data Availability

Available upon reasonable request.

## Supplementary Materials

The following supporting information can be downloaded at: https://www.mdpi.com/article/10.3390/jcm14103567/s1. Supplementary Document S1: Data Protection Assessment German; Supplementary Document S2: Data Protection Assessment English Translation; Supplementary Document S3: Informed Patient Consent German; Supplementary Document S4: Informed Patient Consent German English Translation; Video S1: Transversal Pelvic View after SI Screw Placement; Video S2: Sagittal Pelvic View after SI Screw Placement.

## Abbreviations

The following abbreviations are used in this manuscript:

2D	two-dimensional
3D	three-dimensional
CT	computed tomography
DICOM	digital imaging and communications in medicine
FEA	finite element analysis
HU	Hounsfield Unit
MSCT	multislice computed tomography
SI	sacroiliac
