# Supplementary material for: Ramadanov–Zabler Safe Zone for Sacroiliac Screw Placement: A CT-Based Computational Pilot Study
Source: J Clin Med. 2025 May 20;14(10):3567. doi: 10.3390/jcm14103567 (PMC12112452; doi:10.3390/jcm14103567)
Supplement: Supplementary file 1 [file jcm-14-03567-s001.zip › Supplementary Document S1 - 20250327_Data_Protection_Assessment_German.pdf]

Datenschutzrechtliche Bewertung

Universitätsklinikum Brandenburg an der Havel

Bewertung durch den Datenschutzbeauftragten anhand der bis zum

27.03.2025

eingereichten Unterlagen zur Durchführung der Studie/des Forschungsprojektes:

Ramadanov-Zabler Safe Zone for Sacroiliac Screw Placement: A CT-Based Computational Study (Ramadanov-Zabler-Sicherheitszone für die Platzierung von Iliosakralschrauben: Eine CT-basierte computergestützte Studie)

Verantwortliche Stelle(n)/Klinik(en)/Träger:

Universitätsklinikum Brandenburg GmbH
Zentrum für Orthopädie und Unfallchirurgie
Hochstr 29
14770 Brandenburg an der Havel

Antragsteller/Studienleiter:

Dr. med. Nikolai Ramadanov

Fokus der datenschutzrechtlichen Bewertung:

Die Grundsätze für die Verarbeitung personenbezogener Daten gem. Art. 5 DSGVO wurden beachtet.

Die Erklärung auf welcher Rechtsgrundlage die Daten erfasst und verarbeitet werden, liegt vor.

Das Dokumentationsverfahren (Angaben der zu erfassenden Daten, wer erfasst und bearbeitet die Daten, Löschung der Daten) wurde beschrieben.

Teilnehmerinformation und Teilnehmereinwilligung erfüllen die Vorgaben der DSGVO (Art. 12-14).

Angaben zum Ablauf der Auswahl und Ansprache der Forschungsteilnehmer sind gegeben.

Das Verfahren zum Schutz der Geheimhaltung der Daten und Dokumente wurde beschrieben und vorgelegt.

Angaben zu den technisch-organisatorischen Maßnahmen lt. Stand der Technik wurden beschrieben und sind vorhanden.

Der Umgang mit Publikationen wurde beschrieben.

Kooperationspartner/Sponsoren/Dienstleister sind benannt.

Eine Übermittlung an Dritte, außerhalb der EU bzw. auch außerhalb von Ländern für die die Europäische Kommission einen Angemessenheitsbeschluss gefasst hat, ist nicht vorgesehen.

Es gibt hinsichtlich der vorgelegten und beschriebenen Studie:

- bei Einhaltung der gegebenen Auflagen keine datenschutzrechtlichen Bedenken

Hinweise/Auflagen:

- Der Kooperationspartner ist in den Antragsdokumenten mit zu benennen.
- Es ist ein Auftragsverarbeitungsvertrag (AVV) oder eine Vereinbarung zur Regelung gemeinsamer Verantwortlichkeit mit dem Kooperationspartner abzuschließen.

Karsten Sydow
Datenschutzbeauftragter (DSB)
des Universitätsklinikums Brandenburg

Tel.: 03381 41 2115
Email: dsb@uk-brandenburg.de
Anschrift: Universitätsklinikum Brandenburg GmbH; DSB; 14770 Brandenburg Hochstraße 29
