# Supplementary material for: Ramadanov–Zabler Safe Zone for Sacroiliac Screw Placement: A CT-Based Computational Pilot Study
Source: J Clin Med. 2025 May 20;14(10):3567. doi: 10.3390/jcm14103567 (PMC12112452; doi:10.3390/jcm14103567)
Supplement: Supplementary file 1 [file jcm-14-03567-s001.zip › Supplementary Document S2 - Data_Protection_Assessment_English_Translation.docx]

# Data Protection Assessment (Translation)

Assessment by the Data Protection Officer based on the documents submitted up to:

March 27, 2025

Study/Research Project:

Ramadanov-Zabler Safe Zone for Sacroiliac Screw Placement: A CT-Based Computational Study

Responsible Institution(s)/Clinic(s)/Carrier:

University Hospital Brandenburg GmbH
Center for Orthopedics and Trauma Surgery
Hochstr 29
14770 Brandenburg an der Havel

Applicant/Study Director:

Dr. med. Nikolai Ramadanov

## Focus of the Data Protection Assessment

• The principles for processing personal data in accordance with Art. 5 GDPR have been observed.

• The legal basis on which the data is collected and processed is provided.

• The documentation procedure (details of data to be collected, who collects and processes the data, deletion of the data) is described.

• Participant information and consent fulfill the requirements of the GDPR (Art. 12–14).

• Details on the process for selecting and approaching study participants are provided.

• The procedure for safeguarding the confidentiality of data and documents has been described and submitted.

• Technical and organizational measures according to the state of the art have been described and are available.

• The handling of publications has been described.

• Cooperation partners/sponsors/service providers are named.

• Transfer to third parties outside the EU or to countries without an adequacy decision by the European Commission is not planned.

## Conclusion

There are no data protection concerns regarding the submitted and described study, provided the following conditions are met:

• The cooperation partner must be named in the application documents.

• A data processing agreement (DPA) or an agreement on joint responsibility must be concluded with the cooperation partner.

Karsten Sydow
Data Protection Officer (DPO)
University Hospital Brandenburg

Tel.: 03381 41 2115

Address: University Hospital Brandenburg GmbH; DPO; Hochstraße 29, 14770 Brandenburg
