# Supplementary material for: Ramadanov–Zabler Safe Zone for Sacroiliac Screw Placement: A CT-Based Computational Pilot Study
Source: J Clin Med. 2025 May 20;14(10):3567. doi: 10.3390/jcm14103567 (PMC12112452; doi:10.3390/jcm14103567)
Supplement: Supplementary file 1 [file jcm-14-03567-s001.zip › Supplementary Document S3 - Informed Patient Consent German.pdf]

Einwilligungserklärung zur Teilnahme an der Studie

Titel der Studie: Ramadanov-Zabler Safe Zone for Sacroiliac Screw Placement: A CT-Based Computational Study

Sehr geehrter Patient,
Sie werden gebeten, an einer wissenschaftlichen Studie teilzunehmen. Bevor Sie sich entscheiden, möchten wir Sie über die Studie und die Art der Datenerhebung informieren.

1. Studienzweck

Diese Studie dient der Definition einer präzisen Sicherheitszone für die Platzierung von Iliosakralschrauben anhand eines aus CT-Scans des Beckens abgeleiteten 3D-Modells. Ziel ist es, die chirurgische Sicherheit zu verbessern.

2. Welche Daten werden erhoben?

- CT-Aufnahme des Beckens (DICOM-Format)
- Alter und Geschlecht

3. Wie werden meine Daten verarbeitet und geschützt?

- Ihre Daten werden ausschließlich auf gesicherten Servern gespeichert.
- Der Zugriff ist nur autorisierten Forschern gestattet.
- Die CT-Daten werden verschlüsselt übertragen.
- Eine Weitergabe an Dritte erfolgt nicht.

4. Freiwilligkeit und Widerrufsrecht

Ihre Teilnahme ist freiwillig. Sie können Ihre Einwilligung jederzeit ohne Angabe von Gründen widerrufen. In diesem Fall werden Ihre Daten unverzüglich gelöscht.

5. Dauer der Speicherung und Löschung

Ihre Daten werden nach Abschluss der Studie und Veröffentlichung der Ergebnisse gemäß den Vorgaben der Ethikkommission gelöscht.

6. Kontakt

Bei Fragen wenden Sie sich bitte an:
Dr. med. Nikolai Ramadanov (E-Mail: nikolai.ramadanov@gmail.com, Tel.: 0177 740 66 33)

Einwilligungserklärung

Ich habe die Patienteninformation gelesen und verstanden. Ich erkläre mich freiwillig bereit, an der Studie teilzunehmen.

Name des Patienten: [redacted]

Geburtsdatum: [redacted]

Ort, Datum: Brandenburg an der Havel, 10.03.2025

Unterschrift des Patienten: [redacted]

Patienteninformation zur Studie

Titel der Studie: Ramadanov-Zabler Safe Zone for Sacroiliac Screw Placement: A CT-Based Computational Study

Sehr geehrter Patient,
im Rahmen dieser Studie untersuchen wir die optimale Platzierung von Iliosakralschrauben anhand von CT-Bildern. Ihre Teilnahme hilft dabei, chirurgische Verfahren sicherer zu machen.

Ablauf der Studie

- Wir nutzen eine bereits vorhandene CT-Aufnahme Ihres Beckens.
- Es sind keine zusätzlichen Untersuchungen oder Eingriffe erforderlich.
- Ihre Daten werden ausschließlich für wissenschaftliche Zwecke genutzt.

Ihre Rechte

- Sie können jederzeit ohne Angabe von Gründen aus der Studie austreten.
- Ihre Daten werden nur so lange gespeichert, wie es für die Forschung notwendig ist.
- Ihre Identität bleibt jederzeit geschützt.

Haben Sie Fragen?

Bitte wenden Sie sich an Dr. med. Nikolai Ramadanov

(E-Mail: nikolai.ramadanov@gmail.com, Tel.: 0177 740 66 33).
