# Supplementary material for: Ramadanov–Zabler Safe Zone for Sacroiliac Screw Placement: A CT-Based Computational Pilot Study
Source: J Clin Med. 2025 May 20;14(10):3567. doi: 10.3390/jcm14103567 (PMC12112452; doi:10.3390/jcm14103567)
Supplement: Supplementary file 1 [file jcm-14-03567-s001.zip › Supplementary Document S4 -Informed Patient Consent German English Translation.pdf]

Informed Consent to Participate in the Study

Title of the Study: *Ramadanov-Zabler Safe Zone for Sacroiliac Screw Placement: A CT-Based Computational Study*

Dear Patient,
You are being asked to participate in a scientific study. Before you decide, we would like to inform you about the study and the type of data being collected.

1. Purpose of the Study

This study aims to define a precise safety zone for the placement of iliosacral screws based on a 3D model derived from pelvic CT scans. The goal is to improve surgical safety.

2. What Data Will Be Collected?

- CT scan of the pelvis (DICOM format)
- Age and gender

3. How Will My Data Be Processed and Protected?

- Your data will be stored exclusively on secure servers.
- Access is granted only to authorized researchers.
- The CT data will be transmitted in encrypted form.
- There will be no disclosure to third parties.

4. Voluntariness and Right of Withdrawal

Your participation is voluntary. You can withdraw your consent at any time without providing reasons. In such a case, your data will be deleted immediately.

5. Duration of Storage and Deletion

Your data will be deleted after the conclusion of the study and publication of the results, in accordance with the guidelines of the ethics committee.

6. Contact

If you have any questions, please contact:
Dr. Nikolai Ramadanov
Email: nikolai.ramadanov@gmail.com
Phone: +49 177 740 66 33

Consent Form

I have read and understood the patient information. I voluntarily agree to participate in the study.

Name of the patient: XXXXX
Date of birth: XXXX
Place, date: Brandenburg an der Havel, XXXXX
Signature of the patient: ________________________________

Patient Information for the Study

Title of the Study: *Ramadanov-Zabler Safe Zone for Sacroiliac Screw Placement: A CT-Based Computational Study*

Dear Patient,
In this study, we are investigating the optimal placement of iliosacral screws based on CT images. Your participation will help make surgical procedures safer.

Study Procedure

- We will use an already existing CT scan of your pelvis.
- No additional examinations or interventions are required.
- Your data will be used exclusively for scientific purposes.

Your Rights

- You can withdraw from the study at any time without providing reasons.
- Your data will only be stored as long as necessary for research purposes.
- Your identity will remain protected at all times.

Do you have any questions?
Please contact Dr. Nikolai Ramadanov
Email: nikolai.ramadanov@gmail.com
Phone: +49 177 740 66 33
